# Risk factors, thromboembolic events, and clinical course of New‐Onset Atrial Fibrillation among COVID‐19 hospitalized patients: A multicenter cross‐sectional analysis in Iran

**DOI:** 10.1002/hsr2.813

**Published:** 2022-10-17

**Authors:** Fatemeh Sadat Rahimi, Siamak Afaghi, Farzad Esmaeili Tarki, Hossein Salehi Omran, Mohammad Hossein Nasirpour

**Affiliations:** ^1^ Department of Surgery, Clinical Research and Development Center, Shahid Modarres Hospital Shahid Beheshti University of Medical Sciences Tehran Iran; ^2^ Research Institute of Internal Medicine, Shahid Modarres Hospital Shahid Beheshti University of Medical Sciences Tehran Iran; ^3^ Department of Biomedical Sciences, College of Medicine Florida State University Tallahassee Florida USA

**Keywords:** COVID‐19, New‐Onset Atrial Fibrillation, thrombosis

## Abstract

**Background and Aims:**

We focused on determining the risk factors, thromboembolic events, and clinical course of New‐Onset Atrial Fibrillation (NOAF) among hospitalized coronavirus disease (COVID‐19) patients.

**Methods:**

This retrospective study was conducted in the major referral centers in Tehran, Iran. Of 1764 patients enrolled in the study from January 2020 until July 2021, 147 had NOAF, and 1617 had normal sinus rhythm. Univariate and multivariate Logistic regressions were employed accordingly to evaluate NOAF risk factors. The statistical assessments have been run utilizing SPSS 25.0 (SPSS) or R 3.6.3 software.

**Results:**

For the NOAF patients, the age was significantly higher, and the more prevalent comorbidities were metabolic syndrome, heart failure (HF), peripheral vascular disease, coronary artery disease, and liver cirrhosis. The multivariate analysis showed the established independent risk factors were; Troponin‐I (hazard ratio [HR] = 3.86; 95% confidence interval [CI] = 1.89−7.87; *p* < 0.001), HF (HR = 2.54; 95% CI = 1.61−4.02; *p* < 0.001), bilateral grand‐glass opacification (HR = 2.26; 95% CI = 1.68−3.05; *p* = 0.002). For cases with thromboembolic events, NOAF was the most important prognostic factor (odds ratio [OR] = 2.97; 95% CI = 2.03−4.33; *p* < 0.001). While evaluating the diagnostic ability of prognostic factors in detecting NOAF, Troponin‐I (Area under the curve [AUC] = 0.85), C‐Reactive Protein (AUC = 0.72), and d‐dimer (AUC = 0.65) had the most accurate sensitivity. Furthermore, the Kaplan‐Meier curves demonstrated that the survival rates diminished more steeply for patients with NOAF history.

**Conclusion:**

In hospitalized COVID‐19 patients with NOAF, the risk of thromboembolic events, hospital stay, and fatality are significantly higher. The established risk factors showed that patients with older age, higher inflammation states, and more severe clinical conditions based on CHADS2VASC‐score potentially need subsequent preventive strategies. Appropriate prophylactic anticoagulants, Initial management of cytokine storm, sufficient oxygen support, and reducing viral shedding could be of assistance in such patients.

## 1 INTRODUCTION

The new coronavirus disease (COVID‐19) pandemic, precipitated by severe acute respiratory syndrome coronavirus‐2 (SARS‐CoV‐2),^1^ enormously affecting worldwide communities, has resulted in exceeding 565 million afflicted individuals and over 6 million mortalities as of late July 2022.^2^ The most prevalent clinical symptoms are acute respiratory distress syndrome (ARDS) and Interstitial pneumonia.^3^, ^4^, ^5^, ^6^ Also, the cardiovascular system has been reported to be frequently affected in COVID‐19 patients, and those with cardiovascular involvement are facing a greater risk of worse outcomes.^7^ Atrial fibrillation (AF) is a major etiology for embolism and stroke, particularly if it is not treated with anticoagulative therapy as a preventive stroke measure.^8^ Acute respiratory infections are shown to be represented as one of the risk factors of the new‐onset or recurring AF Stimulation of the sympathetic nervous system, hypoxia, dehydration, electrolyte abnormalities, metabolic dysfunction, and of course, myocardial injuries and inflammation, as consequences of viral pneumonia, increase the likelihood of New‐Onset Atrial Fibrillation (NOAF) and following thromboembolic events.^8^, ^9^, ^10^ It is well understood that the thrombogenic phenomena in AF are not confined to local causes such as defective atrial contraction or stasis. In addition, a generalized hypercoagulative condition has been postulated.^11^ This raises the possibility that additional procoagulant and proinflammatory conditions, such as COVID‐19 pneumonia, might synergistically influence cardiovascular mishaps.^8^, ^11^ Moreover, the virus SARS‐CoV‐2, regardless of causing NOAF, might increase susceptibility to thrombotic illness in both the atrial and venous circulations due to stasis, endothelial dysfunction, platelet activation, and severe inflammation.^12^ Notwithstanding, it is currently uncertain whether SARS‐CoV‐2 causes hemostatic alterations or, as seen in other viral illnesses, are the product of a cytokine cascade that precedes the start of systemic inflammatory response syndrome.^8^, ^13^ Since understanding arrhythmic complications in COVID‐19 is still evolving, we have aimed to assess the clinical characteristics and prognostic factors of NOAF among hospitalized COVID‐19 cases. Also, we evaluated the clinical outcomes of such patients, particularly the thromboembolic events, as a retrospective observational multicenter analysis.

## 2 MATERIAL AND METHODS

### 2.1 Study structure and participants

the present study is a retrospective, multicenter, and observational one performed on the three major COVID‐19 referral centers in Tehran, Iran, affiliated with Shahid Beheshti University of Medical Sciences (Imam Hussein Hospital, Shahid Modarres Hospital, and Shohadaye Tajrish Hospital) from January 2020 until July 2021. All the COVID‐19 patients have been admitted based on the COVID‐19 clinical criteria, approved by the World Health Organization,^14^ and were subsequently verified by real‐time reverse transcriptase‐polymerase chain reaction tests for SARS‐CoV‐2 RNA based on the pharyngeal swabs or lower respiratory tract aspirates.^15^ The present research has been given ethical approval by the ethics institutional reviewing board of Shahid Beheshti University of Medical Sciences, Tehran, Iran (Ethical code: IR. SBMU. RETECH. REC.1399.049). The requirement for obtaining written informed consent has been waived.

### 2.2 Data collection

Patients' information was gathered and analyzed from the hospital medical records and was comprised of demographical characteristics, clinical course during hospitalization, prior medical history, therapy, and in‐hospital outcome. The data were double‐checked independently by four medical researchers. Laboratory tests have been conducted upon admission to the hospital and during the hospitalization period when clinically indicated. Lung spiral computed tomographic scan, and echocardiography have been carried out upon clinical indications.

### 2.3 NOAF definition

The NAOF was defined as the first occurrence of AF upon or during admission in the hospital wards or intensive care units (ICU) in those hospitalized with COVID‐19 and did not have any document or self‐statement regarding previous neglected or managed AF The report of NOAF was conducted in each of the following circumstances: (1) AF lasting for more than 1 h in the electrocardiogram, (2) AF with any duration of existence required cardioversion, (3) AF for which anticoagulation therapeutics were initiated based on CHA2DS2–VASc score criteria. Routine electrocardiogram, pulse rate, and oxygen saturation monitoring were conducted for all patients hospitalized in either wards or ICUs in our mentioned centers. The nursing alarm system report performed the initial suspicion of arrhythmia (nurse/patient ratio: 1/3). The final diagnosis of NOAF, requirement for cardioversion, and initiation of anticoagulants were considered and judged by skilled cardiologists, intensivists, or emergency medical professionals. Further, we excluded patients receiving each of the following high‐risk drugs leading to NOAF based on literature^16^: (1) Any antineoplastic medication (we have excluded patients with malignancy), (2) high‐dose corticosteroids with a dosage of ≥7.5 mg/day equivalents with prednisone,^17^ (3) Cardiovascular medications of adenosine, dobutamine, or milrinone, (4) Opiates, cannabis, or methamphetamines.

### 2.4 Other definitions

The presence of AF and its subtypes were defined based on the recent AF guidelines. NOAF was described as the presence of an AF at ECG throughout the stay in the hospital that was not present upon admission.

Since there was not an organized and common internationally established decision on the administration of prophylactic anticoagulant therapy for hospitalized COVID patients during the time of performing this essay, we defined Prophylactic anticoagulation as administration of heparin‐based anticoagulant components with a prophylactic dose for whom included one of the following criteria upon hospitalization: (1) Noticeably elevated d‐dimer levels in primary laboratory findings, (2) being bed‐ridden or paralyzed, and (3) Clinical/radiological findings are suggestive of ARDS.

Since alcohol consumption has been shown as an independent factor for NOAF incidence, we added this variable in our history with the definition of >30 cc/day in men and >20 cc/day in women as the remarkable alcohol use.^18^

### 2.5 Statistical analysis

The patients were categorized based on whether they were affected by NOAF or not as a comparison. A total of 1764 patients (147 with NOAF and 1617 normal sinus rhythm [control group]) were evaluated in the analysis. We attempted to base most of this study's analytical and interpretational works on a guideline developed by Assel et al.^19^ The Shapiro‐Wilk test was used to check the normal distribution of the data. The categorical data have been provided as numbers (%) and compared employing the *χ* ^2^ test or Fisher's exact test; Continuous data having normal distribution have been provided as mean (standard deviation) then compared employing the independent *t*‐test or the Mann−Whitney *U*‐test when needed. The Wilcoxon test analyzed the skewed variables reported by medians and interquartile ranges. A Cox regression analysis was conducted to determine the factors substantially related to an elevated risk of NOAF. Moreover, for the Cox regression analysis, the number of occurrences restricted the number of variables. The variables chosen for the ultimate regression model were, in fact, the ones with more clinical significance based on the primary comparative analysis. The variables taken into account for the Cox regression model were age, body mass index (BMI), metabolic syndrome, dyslipidemia, diabetes, hypertension, heart failure (HF), chronic obstructive pulmonary diseases (COPD), chronic kidney disease (CKD), coronary artery diseases (CAD), CURB‐65 score, CHA_2_DS_2_VAS_C_ Score, creatinine, albumin, troponin‐I, d‐dimer, C‐reactive protein, Erythrocyte sedimentation rate, serum ferritin, bilateral ground‐glass opacification (GGO), diffuse lung infiltration, and pleural infusion. To investigate the risk factors associated with thromboembolic events among patients with NOAF, a multivariable logistic regression model has been utilized. The Hosmer‐Lemeshow test has been used to assess the fit of the multivariable logistic regression. To avoid overfitting the model, five variables were chosen for multivariable analysis according to clinical limitations and prior findings, with a focus on thrombotic events associated with mortality. Hence, history of NOAF, age, BMI, d‐dimer, and the presence of ARDS have been the six variables selected for the multivariable logistic regression model. We have also plotted Kaplan−Meier curves for within‐hospital death stratified by the occurrence of AF during hospitalization. For the sake of these curves, individuals discharged from the hospital were deemed to have survived because our follow‐up only included the duration of the hospitalization. In all analytical measures, statistical significance was defined as a two‐sided *α* of less than 0.05. The statistical assessments have been run utilizing SPSS 25.0 (SPSS) or R 3.6.3 software.

## 3 RESULTS

### 3.1 Demographic, clinical, laboratorial, and radiological characteristics

**Figure 1:**
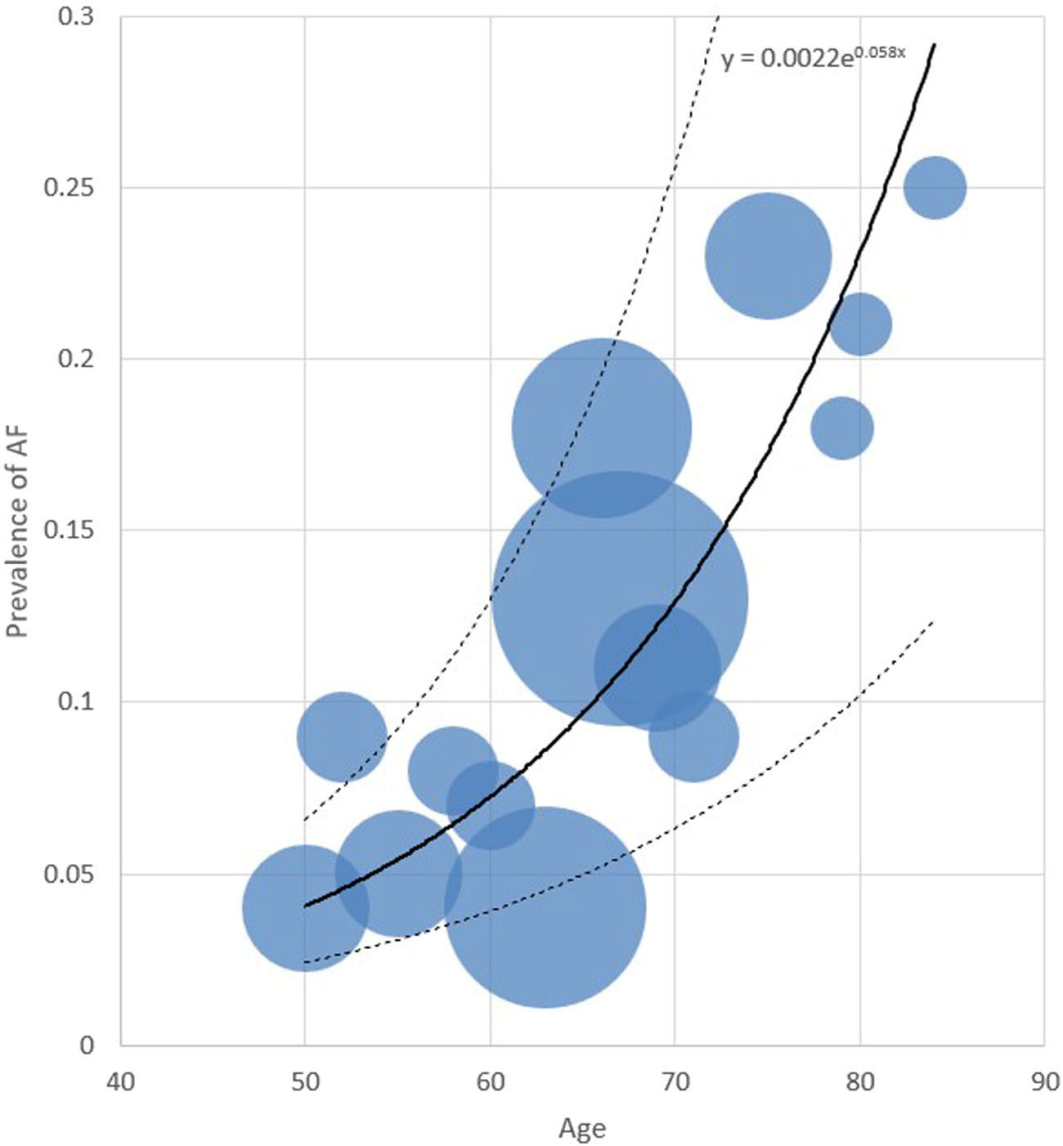
The relation between prevalence of atrial fibrillation and age in patients hospitalized with COVID‐19. The continuous bold line shows the main trendline of how the data are spread throughout the chart. And the two dashed lines above and under the main line show the trendlines of data one standard deviation higher and lower, respectively.

**Figure 2:**
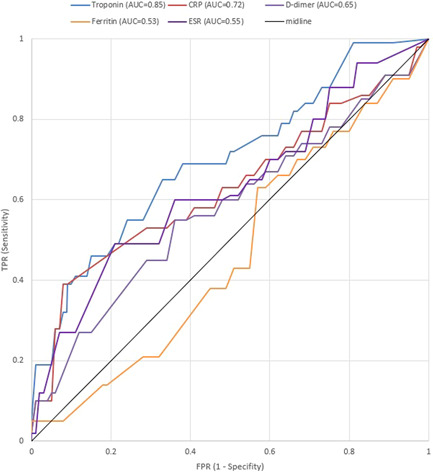
The ROC curves drawn for the five main laboratory markers playing a diagnostic role in detecting AF The Area Under the Curve (AUC) for each of these laboratory markers is a representative measure of the ability of that marker to make a distinction. AF, atrial fibrillation; ROC, receiver operating characteristic.

A comprehensive list of patients' characteristics upon admission has been shown comparatively in Table 1. The age with an average of 66.6 years among all patients was found to have an average of 70.7 years in the NOAF cohort and 66.2 years within the control cohort (*p* < 0.001). As displayed in Figure 1, for the cases in the NOAF cohort, with the increase in age, the prevalence of AF has increased as well, taking a somewhat exponential trend. This means that the prevalence of AF patients might most probably rise as their age increases. Moreover, the total age distribution of all patients is illustrated by the size of bubbles scattered through the diagram. Most of the patients in both study groups have been of the male gender (76.1% vs. 76.9%; *p* = 0.81). The mean BMI has been recorded to be 28.9 in the NOAF group and 27.7 in the control group (*p* = 0.05). The rate of alcohol consumption has been similar in both study groups (*p*  = 0.65). However, the active smoking condition reported as pack‐year had an average of 9.4 years in the NOAF cohort, which was markedly greater than 8.2, the average of the control group (*p* < 0.001). As Table 1 shows, hypertension was found to be the most prevalent comorbidity in both groups (50.3% of the NOAF group and 34.9% of the control group; *p* < 0.001). Prevalence of metabolic syndrome, hypertension, diabetes, dyslipidemia, CKD, CAD, and HF was significantly more common among patients with NOAF; however previous transient ischemic attack, valvular heart disorders, peripheral vascular disease, liver cirrhosis, asthma, Infection with Human Immunodeficiency Virus and Hepatitis C Virus have not indicated a statistically noteworthy contrast between the two study groups. Among the history of drug usage before hospitalization, Angiotensin‐Converting‐Enzyme Inhibitors and Angiotensin‐II Blockers have shown to be of statistically greater use among patients of patients group (38.8% vs. 26.3%; *p* = 0.001). Other drugs, including statins, Beta‐blockers, Aspirin, Warfarin, and Oral direct anticoagulants, whilst having more usage among NOAF patients, did not show a statistically significant difference in comparison to the control group. Regarding clinical signs, the mean oral temperature upon admission was found to be 37.5°C for cases in the NOAF group and 36.9°C for cases in the control group; indicating a statistically remarkable contrast between the two study groups (*p* < 0.001); and SpO_2_ without the mask, with an average of 91.1% among all patients, had a mean of 89.8% in the NOAF cohort which was markedly smaller than the 91.3% mean in the control cohort (*p* = 0.004). Also, the NOAF group had significantly more CURB‐65 score (2.8 vs. 2.4; *p* = 0.003) and CHA_2_DS_2_VASC score (2.6 vs. 1.9; *p* < 0.001) comparing to control group upon admission. As an evaluation of inflammatory factors, the patients with NOAF had significantly greater levels of C‐reactive protein (CRP) (*p* < 0.001)., Erythrocyte sedimentation rate (ESR) (*p* = 0.003), serum ferritin (*p* = 0.005), and d‐dimer (*p* < 0.001) upon admission. In the evaluation of kidney function, glomerular infiltration rate (calculated by Chronic Kidney Disease Epidemiology Collabration (CKD‐EPI) equation) showed a 46.3 ml/min average in the NOAF group and 75.5 ml/min average in the control group, which was significantly lower among the NOAF group (*p* < 0.001). Moreover, creatinine levels, with an average of 1.4 mg/dl in the total patient population, had a 1.6 mg/dl mean in the NOAF group, which was markedly higher than the 1.4 mg/dl mean in the control cohort (*p* < 0.001). Troponin‐I levels, with a mean of 0.01 ng/ml in the total patients population, had a 0.07 ng/ml average in the NOAF group, which was markedly greater than the 0.01 ng/ml mean in the control cohort (*p* < 0.001); Also, Brain Natriuretic Peptide (BNP), with an average of 54.6 pg/ml in the total patients' population, showed a 118.4 pg/ml average in the NOAF group which was outstandingly lower than 49.3 pg/ml average in the control group (*p* = 0.006). Additionally, we noted that patients with NOAF had significantly lower levels of Albumin and Magnesium upon hospitalization. Figure 2 portrays the receiver operating characteristic (ROC) curve drawn for the five main laboratory findings that could play a diagnostic role in detecting AF. These ROC curves are, in fact, graphical plots that illustrate the diagnostic ability of multiple binary classifier systems as their discrimination thresholds are being varied. The area under the curve (AUC) for each of these five laboratory markers is a measure of the ability of that classifier to make a distinction. Therefore, the higher the AUC, the better the performance of that marker at distinguishing between having or not having AF. Accordingly, as depicted in this figure, our findings showed that the laboratory marker with the highest AUC is Troponin‐I; thus, it has the best diagnostic performance among all evaluated markers. Other four laboratory findings, including CRP, d‐dimer, ESR, and Ferritin, have been shown to have AUCs equal to 0.72, 0.65, 0.55, and 0.53, respectively. Eventually, in order for radiological comparison, we obtained that NOAF patients suffered from more severe patterns of lung involvement in which Bilateral GGO (66.6% vs. 54.3%; *p* = 0.004), Diffuse lung infiltration (54.3% vs. 49.6%; *p* = 0.001), and pleural effusion (14.2% vs. 6.06% *p* < 0.001) were found significantly more in NOAF group.

**Table 1:** Comparison of demographic, clinical, laboratorial, radiological, and electrocardiographic characteristics of hospitalized COVID‐19 patients upon admission stratified to New‐Onset Atrial Fibrillation (NOAF) and normal sinus rhythm (control) group

Characteristics	Total *n* = 1764	NOAF *n* = 147	Control *n* = 1617	*p* Value
Demographics				
Age, year	66.6 ± 12.1	70.7 ± 14.1	66.2 ± 11.3	**<0.001**
Gender, male	1356 (76.9)	112 (76.1)	1244 (76.9)	0.81
Body mass index, kg/m^2^	27.9 ± 7.3	28.9 ± 8.3	27.7 ± 7.1	0.05
Alcohol user	124 (7)	9 (6.1)	115 (7.1)	0.65
Active smoker	396 (22.4)	41 (27.9)	355 (21.9)	0.09
Pack‐year	8.3 ± 3.8	9.4 ± 4.4	8.2 ± 3.7	**<0.001**
Comorbidities				
Metabolic syndrome	200 (11.3)	31 (21.1)	169 (10.4)	**<0.001**
Hypertension	639 (36.2)	74 (50.3)	565 (34.9)	**0.000**
Diabetes	281 (15.9)	40 (27.2)	241(14.9)	**0.000**
Dyslipidemia	312 (17.7)	35 (23.8)	277 (17.1)	**0.042**
Heart failure	240 (13.6)	44 (29.9)	196 (12.1)	**<0.001**
Coronary artery disease	379 (21.5)	47 (31.9)	332 (20.5)	**0.004**
Prior stroke/transient ischemic attack	110 (6.2)	14 (9.5)	96 (5.9)	0.08
Valvar heart disorders	196 (11.1)	19 (12.9)	177 (10.9)	0.46
Peripheral vascular disease	52 (2.9)	6 (4.1)	46 (2.8)	0.39
Chronic kidney disease	152 (8.6)	22 (14.9)	130 (8.0)	**0.004**
Liver cirrhosis	73 (4.1)	9 (6.1)	64 (3.9)	0.20
Chronic obstructive pulmonary disease	253 (14.3)	32 (21.8)	221 (13.7)	**0.007**
Asthma	63 (3.6)	7 (4.8)	56 (3.5)	0.41
HIV infection	55 (3.1)	4 (2.7)	51 (3.1)	0.77
HCV infection	17 (0.9)	3(2.0)	14 (0.9)	0.16
Drug history before hospitalization				
ACEI/ARBs	482 (27.3)	57 (38.8)	425 (26.3)	**0.001**
Statin	793 (44.9)	77 (52.4)	716 (44.3)	0.06
Beta‐blockers	285 (16.1)	27 (18.4)	258(15.9)	0.60
Aspirin	180 (10.2)	19 (12.9)	161 (9.9)	0.25
Warfarin	234 (13.3)	21 (14.3)	203 (12.5)	0.54
Oral direct anticoagulants	76 (4.3)	10 (6.8)	66 (4.1)	0.08
Clinical signs				
Respiratory rate, per min	23.5 ± 4.0	23.9 ± 3.2	23.5 ± 4.1	0.24
Systolic blood pressure, mmHg	124.1 ± 15.8	125.6 ± 14.5	124 ± 15.9	0.08
Oral temperature, °C	36.9 ± 1.8	37.5 ± 1.2	36.9 ± 1.6	**<0.001**
SPO_2_ without mask, %	91.1 ± 6.1	89.8 ± 4.4	91.3 ± 6.2	**0.004**
CURB‐65 score	2.4 ± 1.5	2.8 ± 1.1	2.4 ± 1.6	**0.003**
CHA_2_DS_2_VAS_C_ score	1.9 ± 1.5	2.6 ± 1.8	1.9 ± 1.5	**<0.001**
Laboratorial				
White blood count, ×10^9^/L	7.1 (5.8−11)	6.9 (5.5−10.9)	7.2 (6.1−11.9)	0.16
Neutrophil, 10^9^/L	6.1 (4.9−9.7)	5.9 (4.8−9.4)	6.2 (4.9−10.3)	0.24
Lymphocyte, 10^9^/L	0.7 (0.6–1.1)	0.6 (0.5–1.1)	0.7 (0.6–1.3)	0.31
Hemoglobin, g/dl	12.6 (11.1−13.5)	12.5 (11.3−13.8)	12.8 (11.6−13.4)	0.14
Platelet, 10^9^/L	228 (120−298)	225 (135−271)	231 (127−301)	0.33
Alanine transaminase, U/L	30 (24−35)	30 (24−37)	30 (22−34)	0.61
Aspartate aminotransferase, U/L	43 (35−50)	42 (39−53)	45 (36−49)	0.38
Alkaline phosphatase, IU/L	94 (50−106)	97 (53−128)	92 (49−106)	0.09
INR	1.0 (1.0–1.3)	1.0 (1.0–1.4)	1.2 (1.1–1.5)	0.12
Lactate Dehydrogenase, U/L	442 (436−498)	495 (442−516)	435 (421−492)	0.09
Creatinine, mg/dl	1.4 (1.0−1.7)	1.6 (1.0−1.9)	1.4 (1.0−1.6)	**<0.001**
GFR (The CKD‐EPI equation), mL/min	72.6 (48.8−95.2)	46.3 (33.1− 61.9)	75.5 (51.4−97.9)	**<0.001**
Albumin, g/dl	3.1 (2.8−3.6)	3.0 (2.7−3.3)	3.1 (2.7−3.5)	**0.002**
Troponin‐I, ng/ml	0.01 (0.01−0.19)	0.07 (0.02−0.25)	0.01 (0.0−0.09)	**<0.001**
Brain Natriuretic Peptide, pg/ml	54.6 (23.6−183.5)	118.4 (51.3−243.9)	49.3 (19.6−185.3)	**0.006**
d‐dimer, ng/ml	1.46 (0.75−3.05)	2.02 (1.31−3.72)	1.44 (0.77−2.69)	**<0.001**
C‐reactive protein, mg/L	111.4 (44.7−203.5)	123.6 (51.6−215.7)	109.4 (43.5−200.1)	**<0.001**
Erythrocyte sedimentation rate, mm/h	69.0 (40.0−89.0)	72.0 (44.0−93.0)	67.0 (40.0−88.0)	**0.003**
Serum Ferritin, ng/ml	664.0 (342.0−955.0)	697 (361.0−1614.0)	653.0 (291.0−941.0)	**0.005**
Electrolytes				
Sodium, mEq/L	139 (136−141)	136 (134−140)	139 (136−143)	0.13
Potassium, mmol/L	3.9 (3.7−4.6)	4.0 (3.7−4.3)	4.0 (3.7−4.8)	0.46
Calcium, mg/dl	8.6 (7.9−9.6)	8.5 (7.9−8.9)	8.6 (8.0−9.7)	0.14
Magnesium, mg/dl	1.8 (1.7−2.0)	1.7 (1.6−1.9)	1.9 (1.7−2.1)	**0.013**
Radiological				
Bilateral ground glass opacification	977 (55.4)	98 (66.6)	879 (54.3)	**0.004**
Diffuse lung infiltration	656 (37.2)	73 (49.6)	583 (36.0)	**0.001**
Pleural effusion	119 (6.7)	21 (14.2)	98 (6.06)	**<0.001**
Heart rate, beat/min	88.6 ± 13.7	90.5 ± 17.3	88.4 ± 13.3	0.07
P−R interval, ms	153.9 ± 10.4	155.1 ± 13.8	153.8 ± 10.1	0.15
QTC interval, ms	412.5 ± 13.6	414.4 ± 15.2	412.3 ± 13.4	0.07
QRS interval, ms	85.8 ± 9.3	87.0 ± 13.1	85.7 ± 8.9	0.10

*Note*: The *p*‐values shown in bold are indicative of having statistical significance (*p*‐values smaller than 0.05).

Abbreviations: ACEI, Angiotensin‐Converting‐Enzyme Inhibitors; ARBs, Angiotensin‐II Blockers; CKD, chronic kidney disease; CKD‐EPI, Chronic Kidney Disease Epidemiology Collaboration; GFR, glomerular infiltration rate; HCV, Hepatitis C Virus; HIV, Human Immunodeficiency Virus; QRS Interval, QRS complex interval; QTC Interval, corrected QT interval.

### 3.2 Within‐hospital events

Among the administered therapeutics mentioned in Table 2, the followings were significantly utilized more for NOAF patients during the hospitalization: first, steroids (90.5% vs. 70.6%; *p* < 0.001), secondly, Antibiotic therapy by Azithromycin (35.4% vs. 25.9%; *p* = 0.013) and/or Ceftriaxone (23.1% vs. 15.9%; *p* = 0.025), and thirdly oxygen support with FiO_2_ ≥ 50 (%33.3% vs. 24.1%; *p* = 0.014). Moreover, whilst hospitalization we found the following thromboembolic events occurs considerably more in NOAF patients comparing to control group: Cerebral infarction (1.4% vs. 0.2% *p* < 0.001), and Pulmonary embolism (14.3% vs. 7.5%; *p* = 0.004). Also, HF (7.5% vs. 2.6%; *p* = 0.001), and unfavorable bleeding events (26.5% vs. 20.7%; *p* = 0.004) were significantly found more among NOAF patients. However, no statistically significant contrast was noted between the prevalence of myocardial infarction between the two groups (*p* = 0.58). Additionally, the NOAF patients significantly suffered more form hospitalization period (16.5 days vs. 9.4 days; *p* < 0.001), and ICU admission (19.7% vs. 11.2%; *p* = 0.002).

**Table 2:** Therapeutics and clinical outcome among hospitalized COVID‐19 patients upon admission stratified to New‐Onset Atrial Fibrillation (NOAF) and normal sinus rhythm (control) group

Characteristics	Total *n* = 1764	NOAF *n* = 147	Control *n* = 1617	*p* Value
In hospital therapies				
Steroids	1274 (72.2)	133 (90.5)	1141 (70.6)	**<0.001**
Lopinavir/ritonavir	1542 (87.4)	132 (89.8)	1410 (87.2)	0.36
Azithromycin	471 (26.7)	52 (35.4)	419 (25.9)	**0.013**
Ceftriaxone	292 (16.5)	34 (23.1)	258 (15.9)	**0.025**
Remdesivir	1306 (74.0)	117 (79.6)	1189 (73.5)	0.10
Tocilizumab	60 (3.4)	9 (6.1)	51 (3.1)	0.06
Prophylactic anticoagulant	523 (57.0)	62 (42.2)	944 (58.4)	0.16
Therapeutic anticoagulants	482 (27.3)	45 (30.6)	437 (27.0)	0.35
Oxygen support with FiO_2_ < 50%	511 (28.9)	35 (23.8)	476 (29.4)	0.15
Oxygen support with FiO_2_ ≥ 50%	440 (24.9)	49 (33.3)	391 (24.1)	**0.014**
Intubation	243 (13.8)	15 (10.2)	228 (14.1)	0.19
Clinical outcomes				
Heart failure	54 (3.1)	11 (7.5)	43 (2.6)	**0.001**
Myocardial infarction	62 (3.5)	4 (2.7)	58 (3.6)	0.58
Cerebral infarction	6 (0.3)	2 (1.4)	4 (0.2)	**<0.001**
Pulmonary embolism	143(8.1)	21 (14.3)	122 (7.5)	**0.004**
Bleeding events	374 (21.2)	39 (26.5)	335 (20.7)	**0.004**
ICU‐admission	210 (11.9)	29 (19.7)	181 (11.2)	**0.002**
Length of hospital stay, days	9.9 ± 4.5	16.5 ± 6.2	9.4 ± 3.8	**<0.001**
In‐hospital mortality	129 (7.3)	26 (17.7)	103 (6.4)	**<0.001**

*Note*: The *p*‐values shown in bold are indicative of having statistical significance (*p*‐values smaller than 0.05).

Abbreviation: ICU, intensive care unit.

### 3.3 Within‐hospital mortality

**Figure 3:**
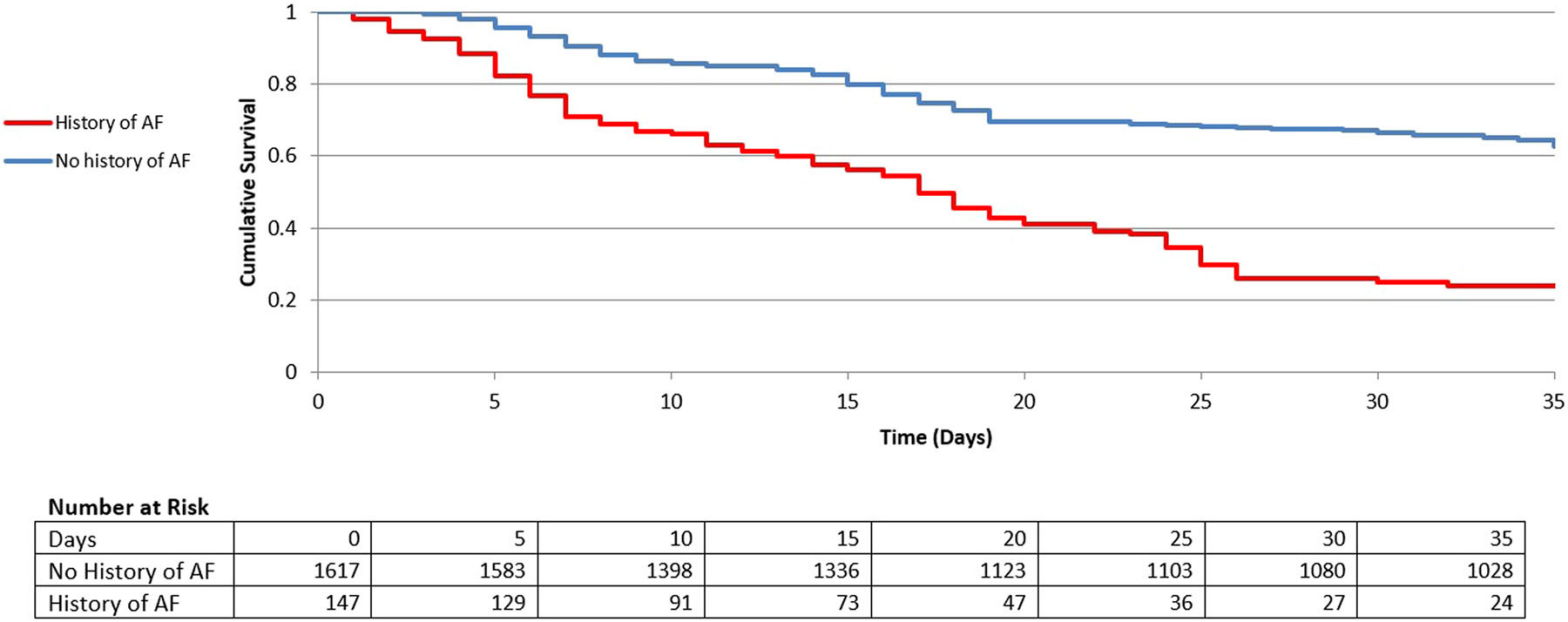
Survival rate stratified by the occurrence of New‐onset Atrial Fibrillation in COVID‐19 patients by the Kaplan–Meier estimator. The survival is approximated based on the days since hospital admission. The hospital‐discharged patients were considered as survived in this analysis.

Eventually, in‐hospital mortality, as the ultimate adverse outcome, was recorded for 17.7% of patients in the NOAF group, while for only 6.4% of individuals within the control group (*p* < 0.001). Figure 3 is the Kaplan−Meier curve for the two main study groups. It is a visual representation of the probability of death as the ultimate adverse outcome at the respective time interval of hospitalization. This curve could give one a clear idea of the survival function of the patients, visualizing the probability that a patient would survive beyond a certain time. As observed in this figure, the survival (approximated based on the days since hospital admission) has diminished much more steeply for patients with a history of AF. In contrast, the patients in the control group seem to be able to keep a higher survival probability as days go by.

### 3.4 Risk factors of NOAF

Table 3 lists the possible risk factors for NOAF among patients hospitalized with COVID‐19, along with the corresponding HR. In fact, the univariate and multivariate analyses performed on these risk factors portray the risk of survival and death for each and every one of them. The univariate analysis of the HR associated with these risk factors showed that for a 5‐year increase in individuals' age, the patients would have 2.79 times more chance of dying in the NOAF cohort in comparison with the control cohort (*p* < 0.001). The multivariate analysis on this resulted in an increased mortality risk of about 1.86 for a 5‐year increase in patients' age (*p* = 0.007). For a 1‐kg/m^2^ increase in BMI, the patients would have a 1.55 times more chance of dying in the NOAF group than in the control group (*p* = 0.014); and the multivariate analysis carried out on this, resulting in an increased mortality risk of about 1.03 for a 1‐kg/m^2^ increase in patients' BMI (*p* = 0.013); implying that there is an almost equal hazard in the two groups. In the presence of Metabolic Syndrome, the patients would have a 3.77 times more chance of dying in the NOAF group compared to the control group (*p* < 0.001). The multivariate assessment performed on this resulted in an increased mortality risk of about 1.66 (*p* = 0.034). If patients have hypertension, they would have a 2.05 times more chance of dying in the NOAF group in comparison with the control group (*p* = 0.001). The multivariate evaluation of this led to an elevated mortality risk of about 1.93 (*p* = 0.020). In the presence of diabetes, the patients would have a 1.85 times more chance of surviving in the control group compared to the NOAF group (*p* = 0.027). The multivariate analysis carried out on this led to statistically insignificant results. If the patients have dyslipidemia, they would have a 2.19 times more chance of dying in the NOAF group as against the control group (*p* = 0.003). The multivariate assessment performed on this brought on statistically insignificant results. In the presence of HF, the patients would be 3.58 times more likely to die in the NOAF cohort as against the Control cohort (*p* < 0.001). The multivariate evaluation conducted on this resulted in heightened mortality risk of about 2.54 (*p* < 0.001). If patients have CAD, they would have 1.65 times more chance of surviving in the control group as against the NOAF group (*p* = 0.027). The multivariate analysis carried out on this led to statistically insignificant results. In the presence of CKD, the patients would have 2.28 times more chance of dying in the NOAF group compared with the control group (*p* < 0.001). The multivariate assessment performed on this resulted in an increased mortality risk of about 1.88 (*p* = 0.001). If patients have COPD, they are 1.37 times more likely to die in the NOAF group than they are in the control group (*p* = 0.021). The multivariate evaluation carried out on this led to statistically insignificant results. For a 1‐point increase in CURB‐65 Score, the patients would have a 1.50 times more chance of surviving in the control group, in comparison with the NOAF group (*p* = 0.014). The multivariate analysis conducted on this, brought on statistically insignificant results. For a 1‐point rise in CHA_2_DS_2_VAS_C_ Score, the patients would be 1.77 times more likely to expire in the NOAF group, compared to the Control group (*p* = 0.004). The multivariate assessment on this resulted in an increased mortality risk of about 1.68 for a 1‐point increase in patients’ CHA_2_DS_2_VAS_C_ Score (*p* = 0.004). If creatinine levels increased by 0.1 mg/dL, the patients would have 1.75 times more chance of dying in the NOAF group as against the control group (*p* < 0.001). The multivariate evaluation of this led to an increased mortality risk of about 1.48 for a 0.1 mg/dl increase in patients' creatinine levels (*p* = 0.002). For a 0.1 g/dl increase in albumin levels, the patients would have a 1.57 times more chance of survival in the control group, in comparison with the NOAF group (*p* = 0.005). The multivariate analysis carried out on this resulted in elevated mortality risk of about 1.42 for a 0.1 g/dl increase in patients' albumin levels (*p*‐value=0.007). If Troponin‐I levels increased by 0.01 ng/ml, the patients would be 4.89 times more likely to die in the NOAF cohort compared to the control cohort (*p* < 0.001). The multivariate evaluation of this led to a heightened mortality risk of about 3.86 for a 0.01 ng/ml increase in patients' Troponin‐I levels (*p* < 0.001). For a 10 ng/ml increase in d‐d‐dimer levels, the patients would have 2.46 times more chance of dying in the NOAF group as against the control group (*p* < 0.001). The multivariate assessment of this resulted in an increased mortality risk of about 1.56 for a 10 ng/ml increase in patients’ d‐dimer levels (*p* = 0.020). If CRP levels rose for 10 mg/ml, the patients would be 2.37 times more likely to die in the NOAF group in comparison with the control group (*p* < 0.001). The multivariate analysis carried out on this led to an elevated mortality risk of about 1.55 for a 10 mg/ml increase in patients’ CRP levels (*p* = 0.011). For a 10 mm/h increase in ESR levels, the patients would have 1.69 times more chance of dying in the NOAF group, compared to the Control group (*p* = 0.001). The multivariate analysis performed on this resulted in an increased mortality risk of about 1.51 for a 10 mm/h increase in patients' ESR levels (*p* = 0.009). If serum ferritin levels increased by 10 ng/ml, the patients would be 1.47 times more likely to expire in the NOAF group than in the control group (*p* = 0.003). The multivariate evaluation of this led to a heightened mortality risk of about 1.13 for a 10 ng/ml increase in patients' serum ferritin levels (*p* = 0.014). If the GGOs were radiologically present, the patients would have 3.87 times more chance of dying in the NOAF cohort as against the control group (*p* < 0.001). The multivariate assessment carried out on this, resulted in an increased mortality risk of about 2.26 (*p* = 0.002). If radiologic evidence of diffuse lung infiltration were present, the patients would be 1.46 times more likely to die in the NOAF group than they would be in the control group (*p* = 0.017). The multivariate analysis performed on this resulted in an increased mortality risk of about 1.48 (*p* = 0.029). If pleural effusion were radiologically present, the patients would have a 1.33 times more chance of survival in the NOAF group compared with the control group (*p* = 0.038). The multivariate analysis conducted on this led to statistically insignificant results.

**Table 3:** Univariate and multivariate analysis of independent risk factors of New‐onset Atrial Fibrillation among patients hospitalized with COVID‐19

Risk factors	Unit/level	Univariable		Multivariable	
		Hazard ratio (95% CI)	*p* Value	Hazard ratio (95% CI)	*p* Value
Age	+5 years	2.79 (1.65−4.71)	**<0.001**	1.86 (1.17−2.95)	**0.007**
Body mass index	+1 kg/m^2^	1.55 (1.09−2.21)	**0.014**	1.03 (0.70−1.53)	**0.013**
Metabolic syndrome	Presence versus absence	3.17 (1.86− 5.41)	**<0.001**	1.66 (1.03−2.67)	**0.034**
Hypertension	Presence versus absence	2.05 (1.18−3.55)	**0.0010**	1.93 (1.10−3.38)	**0.020**
Diabetes	Presence versus absence	1.85 (1.07−3.20)	**0.027**	1.56 (0.93−2.62)	0.09
Dyslipidemia	Presence versus absence	2.19 (1.29−3.71)	**0.003**	1.35 (0.74−2.49)	0.31
Heart failure	Presence versus absence	3.58 (2.35−5.47)	**<0.001**	2.54 (1.61−4.02)	**<0.001**
Coronary artery disease	Presence versus absence	1.65 (1.05−2.57)	**0.027**	1.25 (0.85−1.84)	0.24
Chronic kidney disease	Presence versus absence	2.28 (1.43−3.63)	**<0.001**	1.88 (1.28−2.74)	**0.001**
Chronic Obstructive	Presence versus absence	1.37 (0.97−1.93)	**0.021**	1.30 (0.76−2.22)	0.33
Pulmonary Disease					
CURB‐65 Score	+1 point	1.50 (1.08−2.09)	**0.014**	1.32 (0.93−1.88)	0.11
CHA_2_DS_2_VAS_C_ Score	+1 point	1.77 (1.01−3.10)	**0.004**	1.68 (1.01−2.80)	**0.004**
Creatinine	+0.1 mg/dl	1.75 (1.28−2.38)	**<0.001**	1.48 (1.06−2.06)	**0.002**
Albumin	+0.1 g/dl	1.57 (1.14−2.17)	**0.005**	1.42 (0.96−2.10)	**0.007**
Troponin‐I	+0.01 ng/ml	4.89 (2.29−10.46)	**<0.001**	3.86 (1.89−7.87)	**<0.001**
d‐dimer	+10 ng/ml	2.46 (1.80−3.37)	**<0.001**	1.56 (1.07−2.27)	**0.020**
C‐ reactive protein	+10 mg/L	2.37 (1.58−3.530	**<0.001**	1.55 (1.10−2.19)	**0.011**
Erythrocyte sedimentation rate	+10 mm/h	1.69 (1.21−2.34)	**0.001**	1.51 (1.10−2.07)	**0.009**
Serum Ferritin	+10 ng/ml	1.47 (1.07−2.02)	**0.003**	1.13 (0.790−1.61)	**0.014**
bilateral ground glass opacification	Presence versus absence	3.87 (2.71−5.51)	**<0.001**	2.26 (1.68−3.05)	**0.002**
Diffuse lung infiltration	Presence versus absence	1.46 (1.06−1.99)	**0.017**	1.48 (1.04−2.10)	**0.029**
Pleural effusion	Presence versus absence	1.33 (0.96−1.85)	**0.038**	1.18 (0.81−1.73)	0.38

*Note*: The *p*‐values shown in bold are indicative of having statistical significance (*p*‐values smaller than 0.05).

Abbreviation: CI, confidence interval.

### 3.5 Risk factors of thromboembolic events

As shown in Table 4, to indicate an indisputable impact of NOAF in the occurrence of thromboembolic events, we designed univariate and multivariate analyses to evaluate the possible prognostic factors for thromboembolic events along with the associated OR. Based on univariate analysis, it was found that if the patients have NOAF, they are 5.38 times more likely to develop thromboembolic events (*p* < 0.001). The following prognostic factors of age (OR = 3.46, *p* < 0.001), hospitalization duration (OR = 2.73, *p* < 0.001), ARDS (OR = 2.37, *p*‐value=0.04), CHA_2_DS_2_VAS_C_ score (OR = 2.21, *p* < 0.001), BMI (OR = 1.84, *p* = 0.002), CURB‐65 score (OR = 1.71, *p* = 0.06), d‐dimer levels (OR = 1.44, *p* < 0.001), active cancer (OR = 1.25, *p* = 0.18), CRP (OR = 1.14, *p* = 0.038), intubation (OR = 1.12, *p* = 0.14), and active smoking (OR = 1.00, *p* = 0.72) were respectively assessed as possible prognostic factors of thromboembolic events. Regarding to multivariate analysis, NOAF (OR = 2.97, *p* < 0.001), d‐dimer (OR = 2.68, *p* < 0.001), CHA_2_DS_2_VAS_C_ score (OR = 1.83, *p* = 0.001), hospitalization duration (OR = 1.83, *p* = 0.025), age (OR = 1.26, *p* = 0.033), and BMI (OR = 1.16, *p* = 0.09), have been evaluated as independent predictive factors for prognosis of thromboembolic events. Ultimately, it's concludable that NOAF is the most considerable prognostic factor for the occurrence of thromboembolic events.

**Table 4:** Prognostic factors of thromboembolic events among patients hospitalized with COVID‐19

Characteristic	Univariate		Multivariate	
	OR (CI 95%)	*p* Value	OR (CI 95%)	*p* Value
New Onset Atrial Fibrillation	5.38 (3.40–8.50)	**<0.001**	2.97 (2.03–4.33)	**<0.001**
d‐Dimer, μg/ml	1.44 (0.91–2.28)	**<0.001**	2.68 (1.913–3.75)	**<0.001**
CHA_2_DS_2_VAS_C_ score	2.21 (1.30–3.75)	**<0.001**	1.83 (1.27–2.64)	**0.001**
Hospitalization duration, day	2.73 (1.77–4.21)	**<0.001**	1.83 (1.33–2.51)	**0.025**
Age, year	3.46 (2.12–5.64)	**<0.001**	1.26 (0.83–1.87)	**0.033**
Body Mass Index, kg/m^2^	1.84 (1.23–2.75)	**0.002**	1.16 (0.77–1.75)	0.09
Acute respiratory distress syndrome (ARDS)	2.37 (1.58–3.57)	**0.04**	_	_
CURB‐65 Score	1.71 (1.04–2.80)	0.06	_	_
Active cancer	1.25 (0.89–1.76)	0.18	_	_
C‐reactive protein pg/ml	1.14 (0.72–1.79)	**0.038**	_	_
Intubation	1.12 (0.85−1.48)	0.14	_	_
Active smoking	1.00 (0.76−1.31)	0.72	_	_

*Note*: The *p*‐values shown in bold are indicative of having statistical significance (*p*‐values smaller than 0.05).

Abbreviations: CI, confidence interval; OR, odd ratio.

## 4 DISCUSSION

In this study, we found out that: (1) Factors including age, metabolic syndrome, arterial hypertension, HF, renal dysfunction, and diffused lung involvement acted as independent factors for the NOAF in patients with COVID‐19, (2) Levels of troponin‐I have a more accurate diagnostic role in the detection of NOAF rather than inflammatory factors of CRP, ESR, ferritin, and d‐dimer, (3) NOAF during hospitalization in COVID‐19 patients is an independent predictor of within‐hospital thromboembolic events, (4) NOAF is related to poorer clinical characteristics during the hospital stay, such as more prevalent bleeding episodes and more events in the combined end‐point (embolic events accompanying mortality), (5) And, NOAF has been linked to a lengthier hospital stay. Arrhythmogenic stimuli, such as persistent hypotension, insufficient oxygen supply, or past use of vasopressors, might enhance NOAF while having COVID‐19 disease, particularly in critical illness. According to this viewpoint, NOAF might be both a measure of illness severity and a possible factor in adverse results. In fact, we suggest the connection between COVID‐19 and AF is bilateral. Multiple studies have shown that AF might elevate the severity and incidence of COVID‐19; in turn, the COVID‐19 disease might favor further episodes of AF.^20^, ^21^, ^22^, ^23^ NOAF is rather a common arrhythmia in several diseases, including severe COVID‐19 pneumonia. On the other hand, as we have shown in the study, it is linked to an elevated occurrence of additional problems such as stroke, lengthier hospitalization, and higher hospital‐related costs. As prevention of AF has always been a legitimate clinical objective, several randomized studies have been assessed. It would also be essential to discover NOAF predictors to appropriately monitor the difficult balance between embolic events and the bleeding risks of taking prophylactic medications. Preliminary findings indicate that COVID‐19 cases have hemostatic problems, including disseminated intravascular coagulation.^24^ This complicates therapy since people with both diseases require anticoagulant medication. Previous studies found that the percentage of COVID‐19 cases at hospitals with an established history of AF ranges from 5% to 20%.^20^ In our experience, the high prevalence of NOAF history (147/1764, 8.3%) might have been associated with the higher age of patients (mean age = 66.6 ± 12.1 years). Furthermore, it might be related to our cohort's adherent features, including a high frequency of cardiovascular comorbidities (hypertension = 36.2%, HF = 13.6%). While in our study, within‐hospital NOAF in COVID‐19 cases was reported more prevalently in patients with pre‐existent comorbidities, we also clinically found that NOAF could be found among those without underlying disease. Rather, NOAF has been affected mostly by; First, inflammation biomarkers such as CRP, which have previously been linked to AF; secondly, laboratory biomarkers such as d‐dimer and Troponin‐I, which correspond with the severity of COVID‐19 disease; and thirdly, administering corticosteroids, which, quite apart from potential drug effect, is commonly directed against those exhibiting the greatest hyperinflammation response. Supporting our findings, elevated inflammatory mediating factors such as Tumor Necrosis Factor‐α (TNF‐α), Interleukin‐6, and CRP^25^, ^26^, ^27^, ^28^ as well as the presence of acute metabolic disorders (including hypoxemia, hypo/hyperthermia, and electrolyte abnormalities) have been identified as inducers for AF while being hospitalized for pneumonia.^21^, ^29^ Inflammation, among these elements, has been demonstrated to have a crucial role in the progression of NOAF. It is of note to keep in mind that its effect is independent of conventional risk factors (coronary artery disease and hypertension).^30^, ^31^, ^32^, ^33^ These findings point to a possible molecular connection between inflammatory responses and new atrial arrhythmias in COVID‐19 cases. Furthermore, the existence of bilateral diffuse lung infiltration and GGO in radiological observations was revealed to be one of the greatest independent factors influencing NOAF development in the current study. Because widespread lung infiltration is a predictor of more severe pneumonia, it might be linked to a more pronounced inflammatory status throughout the COVID‐19 clinical course. The pathologic explanation might be cytokine‐induced vasoconstriction, which produces an ischemic state at the pulmonary venous atrial interface, which is where the majority of AFs develop.^26^, ^34^ Aside from the heightened inflammatory state, an upsurge in endogenous catecholamine secretion and also hemodynamic imbalance might contribute to AF When pulmonary inflammation develops, gas exchange is disrupted, resulting in a ventilation/perfusion mismatch and, hence, hypoxemia. Consequently, widespread lung infiltration might induce hypoxemia, which might stand as another cause of NOAF in COVID‐19 patients.

Notwithstanding, it is of essence to note that comparing NOAF patients with the control group of this cohort, the temperature on admission appeared subfebrile, and oxygen saturation reduced but not critically. However, the CURB‐65 Score was moderate‐high, and lung damage had been more significant in the AF group. But the difference between the groups was not that remarkable. Some biomarkers, such as NT‐pro‐BNP, reflected the presence and possible worsening of HF, which was more common in the NOAF group. It could be concludable that patients who were believed to develop AF were older and sicker individuals who were more likely to have AF irrespective of COVID‐19, and subsequently, the impact of COVID‐19 per se on the development of AF could be modest.

## 5 LIMITATIONS

Of note, our study has multiple limitations: For starters, our cohorts were rather comparatively elderly, with an already higher incidence of cardiovascular disease. Because we could not exclude the remaining confounding effect, the predictive function of AF history should be considered as such. Meanwhile, significant clinical and demographic confounding factors were accounted for in the multivariable model.

Second, since we didn't have access to follow‐up data after discharge, we were unable to analyze the occurrence and subsequent implications of NOAF after hospitalization.

Third, in our study, AF was diagnosed using in‐hospital ECG and considered new‐onset if it was not present on admission; however, it is not thoroughly clear whether pre‐hospitalization/pre‐COVID‐19 AF was present, particularly with regard to paroxysmal AF.

Fourth, the unavailability of long‐term ECG monitoring or Holter monitoring for all participants was one of the study's major weaknesses, raising the possibility that silent AF had gone unnoticed.

Fifth, it is the possibility of underdiagnosing ischemic strokes due to the difficulties of conducting brain imaging studies on infected individuals. Sixth, since we excluded the patients with a previous history of high‐risk drugs for developing NOAF to decrease the effect of confounding factors, we did not assess the probable role of in‐hospital medications received upon COVID‐19 admission in our regression model. As data about the potential role of corticosteroid pulse therapy, antibacterial agents as co‐infection management, or antivirals such as Remdesivir on developing arrhythmias specifically regarding NOAF is scarce; it is of the essence for medical researchers to consider these factors for ongoing studies. Seventh, we could not consider all the potential risk factors of NOAF, such as obstructive sleep apnea, in our model regression. Last but not least, one should bear in mind that all the mentioned findings only apply to hospitalized individuals; nonhospitalized COVID‐19 cases may have different determinants of NOAF development and thereby different outcomes.

## 6 CONCLUSIONS

In conclusion, patients with NOAF in the setting of COVID‐19 have a poorer prognosis due to an increased risk of embolic events, hospital stay, intubation, and fatality. The development of AF in individuals with COVID‐19 has ramifications beyond the mere existence of the arrhythmia, and the prognosis in these patients is poorer than that in those with sinus rhythm. Patients with older age, higher inflammation states, and more severe clinical conditions based on CHADS_2_VASC score potentially need subsequent therapeutic strategies. Further research is required to develop approaches that can anticipate the development of new AF to recognize high‐risk individuals, offer early therapy, and thereby reduce the thromboembolic risks of COVID‐19 and AF.

## AUTHOR CONTRIBUTIONS

**Fatemeh Sadat Rahimi**: Conceptualization; data curation; formal analysis; investigation; methodology; visualization; writing − original draft; writing − review and editing. **Siamak Afaghi**: Conceptualization; Data curation; Formal analysis; Investigation; Methodology; Visualization; Writing − original draft; Writing − review and editing. **Farzad Esmaeili Tarki**: Conceptualization; Formal analysis; Methodology; Project administration; Supervision; Validation; Visualization; Writing − original draft; Writing − review and editing. **Hossein Salehi Omran**: Data curation; Methodology; Writing − original draft. **Mohammad Hossein Nasirpour**: Formal analysis; Methodology; Writing − original draft.

## CONFLICT OF INTEREST

The authors declare that no conflict of interest.

## TRANSPARENCY STATEMENT

The lead author Farzad Esmaeili Tarki affirms that this manuscript is an honest, accurate, and transparent account of the study being reported; that no important aspects of the study have been omitted; and that any discrepancies from the study as planned (and, if relevant, registered) have been explained.

## Data Availability

The data that support the findings of this study are available on request from the corresponding author. The data are not publicly available due to privacy or ethical restrictions
